# Complete urethral preservation in robot‐assisted radical prostatectomy: step‐by‐step description of surgical technique

**DOI:** 10.1111/bju.16508

**Published:** 2024-08-27

**Authors:** Tarek Al‐Hammouri, Ricardo Almeida‐Magana, Lazaros Tzelves, Osama Al‐Bermani, Zafer Tandogdu, Jeremy Ockrim, Greg Shaw

**Affiliations:** ^1^ Department of Urology University College London Hospitals London UK; ^2^ Centre of Medical Imaging University College London London UK; ^3^ Department of Targeted Intervention University College London London UK

**Keywords:** Bladder neck‐sparing prostatectomy, stress urinary incontinence, prostate cancer, robot‐assisted surgery, urinary function

**Abbreviations**
BCR: biochemical recurrence
BNP: bladder neck preservation
CUP: complete urethral preservation
FB: fenestrated bipolar
ICR: immediate continence recovery
(RA)RP: (robot‐assisted) radical prostatectomy
UI: urinary incontinence

## Introduction

Robot‐assisted radical prostatectomy (RARP) is one of the treatment options for localised clinically significant prostate cancer [1]. However, postoperative urinary incontinence (UI) affects 4–31% of patients 12 months after surgery and is associated with a reduced quality of life [2]. Several surgical strategies have been described to reduce the incidence of UI, including anterior and posterior reconstruction [3], dorsal venous complex and preperitoneal space sparing (PSS) [4], but no consensus exists on the best method to achieve early return of continence.

**Fig. 1:**
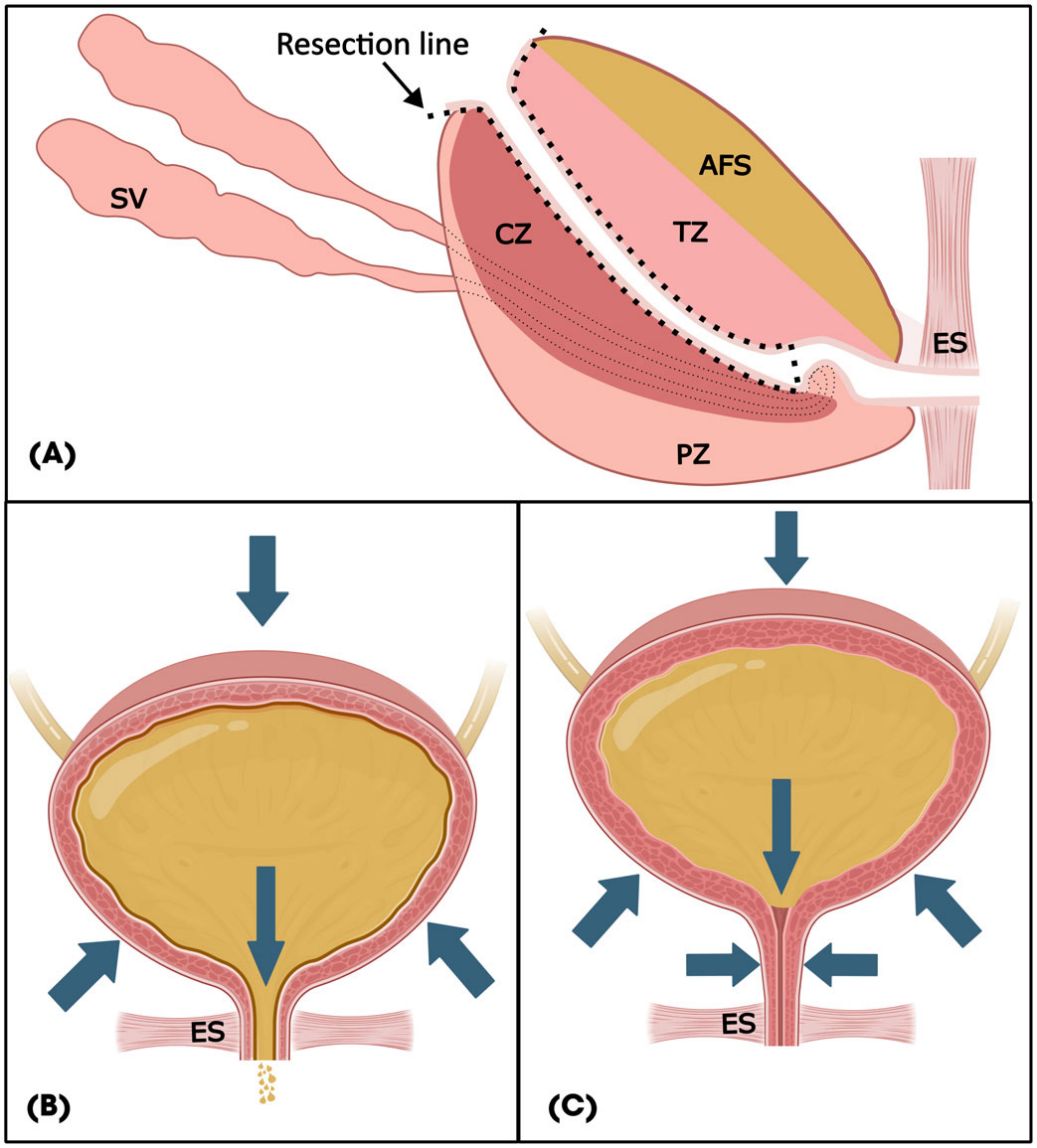
(**A**) A longer segment of the urethra can be preserved when dissected proximally, down to verumontanum. (**B**) In non‐CUP, intra‐abdominal pressure is not transmitted to the shorter urethra and UI can occur. (**C**) CUP allows transmission of intra‐abdominal pressure and coaptation of the longer urethra to prevent UI. AFS, anterior fibromuscular stroma; CZ, central zone; ES, external sphincter; PZ, peripheral zone; SV, seminal vesicles; TZ, transitional zone.

Bladder neck preservation (BNP) aims to safeguard the internal sphincter (lisso‐sphincter), believed to support passive continence, which was recently supported by a systematic review [5]. This effect could be amplified by increasing the length of the spared intraprostatic urethra to achieve coaptation when intra‐abdominal pressure increases [6] (Fig. 1). In fact, a urethral sparing method was described by Tongco et al. [7] in open RPs but was never widely adopted.

The advantages of robotic surgery allow for improved anatomical dissection, to go beyond the standard BNP and dissect the intraprostatic urethra away from the prostatic tissue in a reproducible way. In this paper, we describe the steps and anatomical landmarks to perform the complete urethral preservation (CUP) technique. Our objective was to evaluate the rate of immediate continence recovery (ICR), and present oncological outcomes in a cohort of patients with a minimum 1‐year follow‐up (Video 1).

**Video 1:** Step‐by‐step video description of the CUP technique.

## Patients and Methods

We retrospectively collected data for patients with prostate cancer who underwent RARP with CUP at University College London Hospitals, from June 2021 to August 2022. Surgeries were performed by a single high‐volume urological surgeon (G.S.), and by trainees under supervision, using the da Vinci X/Xi® platform (Intuitive Surgical Inc., Sunnyvale, CA, USA).

A successful CUP was defined as the incision of the urethra at the proximal end of the verumontanum with direct end‐to‐end anastomosis to the membranous urethra. Continence outcomes were collected during clinical follow‐up. ICR was defined as the absence of leakage and the use of zero pads immediately after urethral catheter removal. Biochemical recurrence (BCR) was defined as a PSA level of 0.2 ng/mL at any point after RARP. All data were collected by a dedicated database manager as part of the prospective audit within the quality assurance programme, additional data specific for this project were collected retrospectively by T.A.H., R.A., G.S., L.T. and O.A. Descriptive statistical analysis was performed using R version 4.3.2 (R Foundation for Statistical Computing, Vienna, Austria).

## The CUP Surgical Technique

### Anterior Dissection

**Fig. 2:**
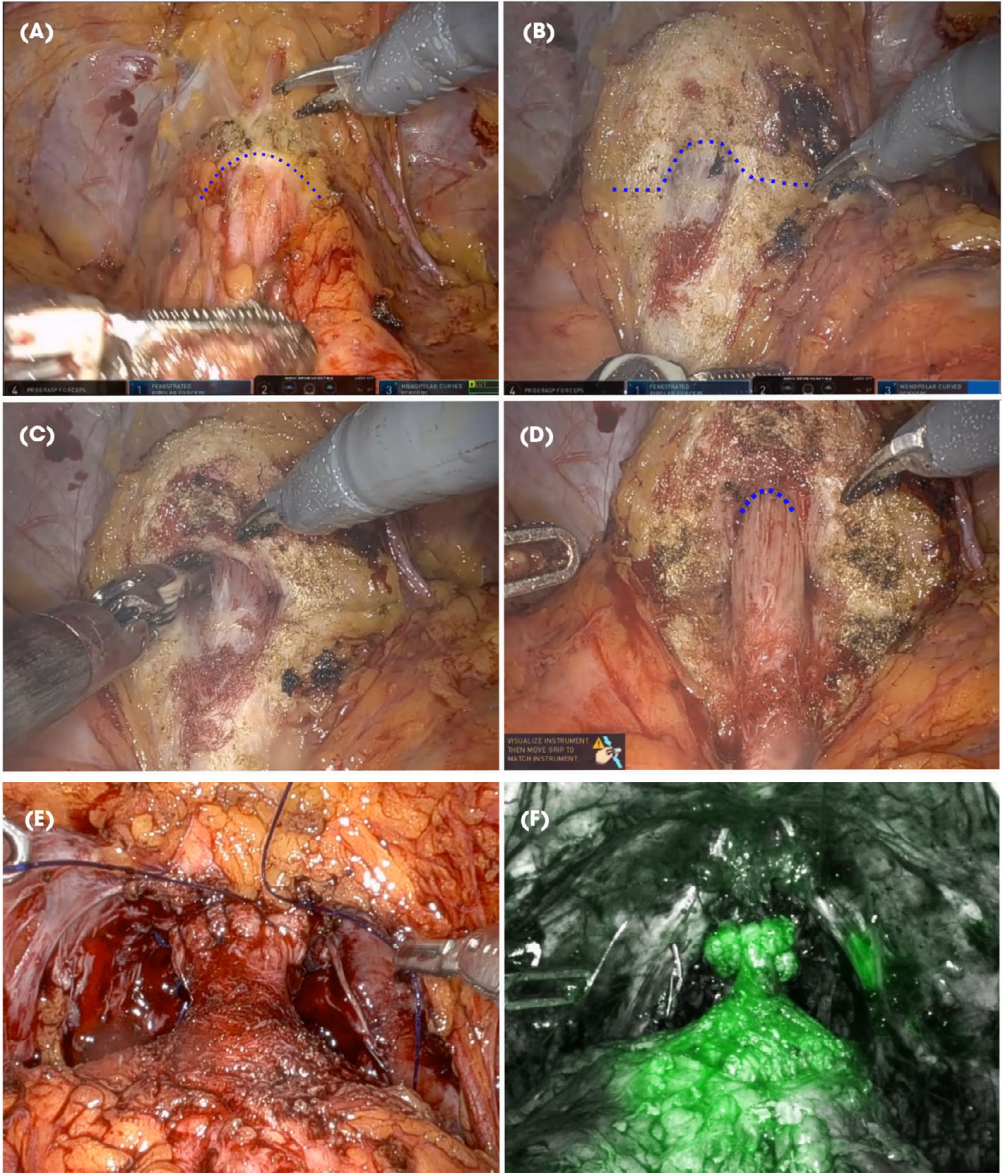
Key steps for CUP. (**A**) Incision at the proximal edge of the puboprostatic ligaments. (**B**) Posterior retraction shows the vertical lines of the urethra. (**C**) Blunt dissection using active opening mechanism of the FB forceps on the sides of the urethra. (**D**) Exposure of the distal end of the urethral incision. (**E**) Final look of anastomosis. (**F**) Indocyanine Green (ICG) image of anastomosis demonstrating vascularity.

Trocar placement is standard for anterior RARP [8]. After developing the Retzius space, removal of pre‐prostatic fat to expose the puboprostatic ligaments, the bladder is retracted cranially and posteriorly using a ProGrasp® instrument (Intuitive Surgical Inc.). An incision is made in the bladder muscular fibres with monopolar scissors proximal to the edge of the puboprostatic ligaments (Fig. 2A). By changing the tension of bladder retraction posteriorly, dissection proceeds along the avascular plane of the lateral vesico‐prostatic junction, where the plane resembles the ‘spine of an open book’ (Fig. 2B). Once the vertical fibres of the urethra are identified, perform blunt dissection along the lateral edges using the active opening of the fenestrated bipolar (FB) forceps (Fig. 2C). Correct alignment is indicated by finding the white avascular plane. The FB forceps can be used to lift the prostate away from the urethra, exposing its anterior surface at the most distal point achievable (Fig. 2D). The urethra appears thin and changes trajectory near the level of the verumontanum.

### Urethral Incision

The anterior urethra is incised horizontally at the most distal point. If needed, the catheter can be used for anterior retraction with the ProGrasp to expose the verumontanum. Otherwise, exert cranial retraction of the bladder and incise the lateral and posterior sides of the urethra. Then divide the posterior urethra while avoiding the tendency to cut proximally, which can create a deficient posterior urethral cuff. Careful ‘hot’ dissection with monopolar scissors is employed to separate the remaining attachments.

Once free, the posterior lip of the urethra is grasped with the FB forceps and gentle cranial retraction is applied. Monopolar electrocautery helps separate the posterior urethra off the prostate, dividing the ejaculatory ducts. If a median lobe is present, this can be carefully dissected off of the urethral stump at this stage. To avoid tearing the urethral stump during retraction, advancement of one jaw of the FB forceps as far as possible across the posterior wall of the urethral stump is effective. The lateral fat pads between the bladder and prostate serve as helpful landmarks. The detrusor slips are then incised until the two vasa are visualised running in the midline.

### Subsequent Steps

Occasional inadvertent buttonholing around the bladder neck or splits to the urethral cuff can be repaired with 3/0 absorbable sutures. The rest of the RARP is completed, including neurovascular bundles and anterior tissue‐sparing techniques. The anastomosis of the urethral ends is performed using 3/0 barbed sutures circumferentially and crossing anteriorly with eight to 10 throws. A single knot is used to snug down the anastomosis.

## Results

**Fig. 3:**
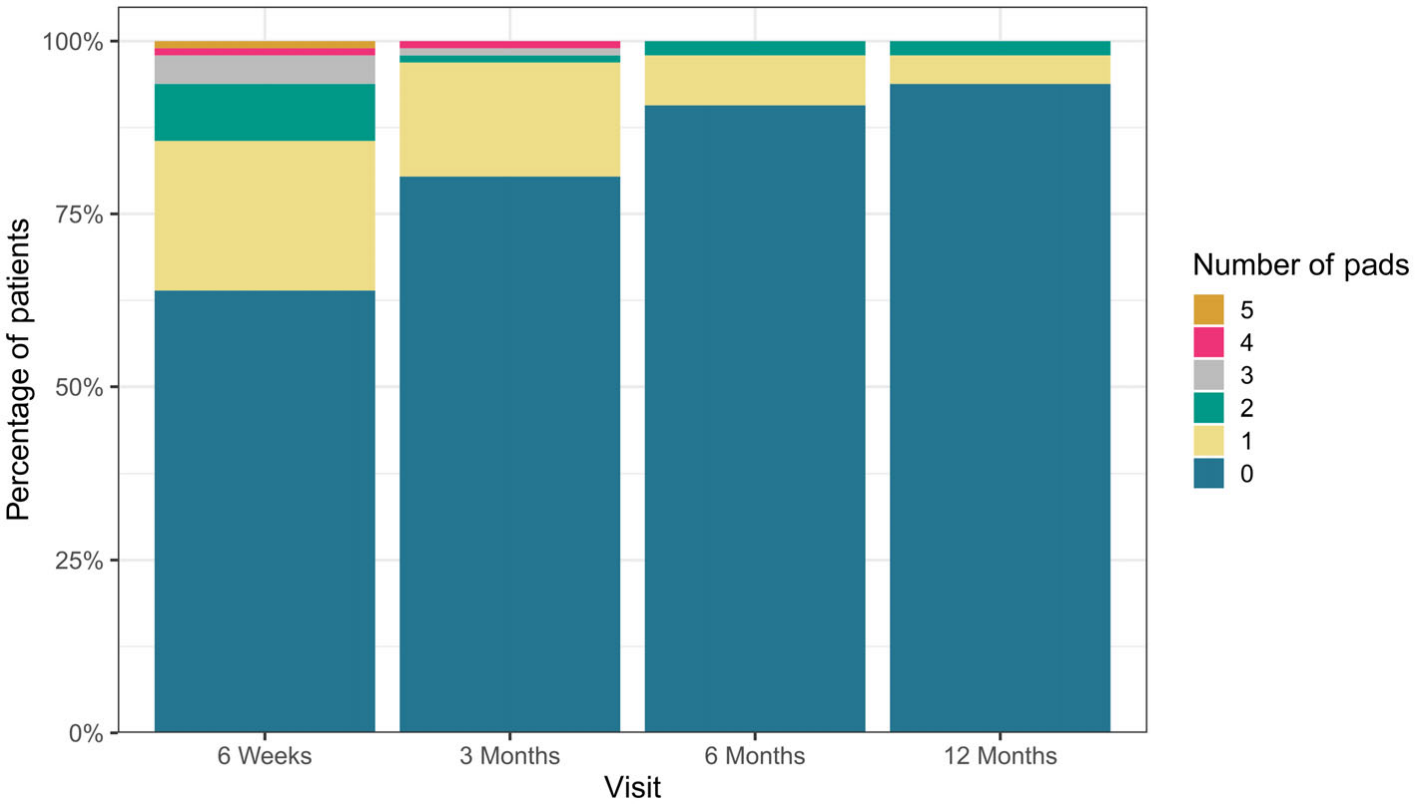
Bar chart showing number of pads at each visit, at 6 weeks 64% of patients report no use of urinary pads increasing to 94% at 12 months after RARP.

A RARP with CUP was performed in 97 patients. Pre‐ and postoperative key patient characteristics are shown in Table 1. Continence outcomes at different visits are shown in Fig. 3. Detailed numbers and percentages can be found in Table S1. All complications were classified as Clavien–Dindo Grade I–II as shown in Table S2.

**Table 1:** Patients characteristics and outcomes.

Characteristic	Value
Number of patients	97
Age, years, median (range)	59 (43.0–76.0)
Body mass index, kg/m^2^, median (range)	27 (20.0–35.0)
Membranous urethral length, mm, median (range)	15 (8.0–22.0)
Presence of median lobe on MRI, *n* (%)	25 (28.1)
PSA level at diagnosis, ng/mL, median (range)	6 (2.7–45.4)
Gland volume measured at MRI, mL, median (range)	30 (16.0–112.0)
Cambridge prognostic group category, *n* (%)	
1	2 (2.1)
2	47 (48.5)
3	14 (14.4)
4	26 (26.8)
5	8 (8.2)
Nerve‐sparing performed, *n* (%)	
No	7 (7.2)
Unilateral	29 (29.9)
Bilateral	61 (62.9)
Training during case, *n* (%)	20 (20.6)
Console time, min, median (range)	100 (45.0–230.0)
Positive margin locations, *n* (%)	22 (22.6)
Apex	5
Circumferential	14
Base	3
BCR at 1 year, *n* (%)	11 (11.3)
Immediate continence after catheter removal, *n* (%)	51 (52.6)

## Discussion

This cohort of patients who underwent RARP with the CUP technique have a high proportion of both ICR and complete continence at 12 months, despite using a strict continence definition. This proportion of ICR is similar to rates (45–69%) previously described with PSS prostatectomy [6]. The CUP technique involves a relatively easy technical modification rather than a major change in approach for those who favour the anterior approach.

Previous descriptions of urethral preservation employed a retrograde approach starting from the membranous urethra at the apex [9]. However, the maximum length of preservable urethra is limited by the natural distal insertion of the ejaculatory ducts at the verumontanum, as shown in Fig. 1A.

In our study, the rate of positive margins, particularly basal prostatic margins, and BCR are comparable to our current practice and published meta‐analysis [10]. Reassuringly no urethral strictures/contractures and only one case of urinary retention was observed.

The learning curve for mastering the technique seems feasible. Approximately 10 supervised cases were adequate for our trainees to perform CUP independently. While these initial results are encouraging, we recognise the need for prospective randomised comparative studies to understand the impact of CUP on continence outcomes. Additionally, it is important to formally evaluate the learning curve to achieve consistent CUP quality.

This technique is not without limitations; we avoid performing CUP in salvage RARP and in patients with a history of BOO surgery, where the bladder neck is deficient. In cases with anterior basal prostate tumours, an oblique approach to the anterior urethra leaves a detrusor cuff and reduces the risk of basal PSM, as described in PSS surgery [11]. In rare cases where MRI locates peri‐urethral tumours, or bladder neck invasion is suspected, we do not perform CUP.

## Conclusion

The CUP technique is a reproducible method to achieve early continence recovery. In this case series, we observed a high rate of ICR, without compromising complications or oncological safety. Future research with randomised cohorts would be essential for validating these encouraging findings.

## Disclosure of Interests

Authors declare they have no conflict of interest.

## Supporting information


**Table S1** Number of UI pads reported by patients at each postoperative visit.
**Table S2** Description of complications reported during follow‐up according to the Clavien–Dindo classification.
