# Supplementary material for: Complete urethral preservation in robot‐assisted radical prostatectomy: step‐by‐step description of surgical technique
Source: BJU Int. 2024 Aug 27;135(1):171–5. doi: 10.1111/bju.16508 (PMC11628903; doi:10.1111/bju.16508)
Supplement: Supplementary file 1 — Table S1 Number of UI pads reported by patients at each postoperative visit. Table S2 Description of complications reported during follow‐up according to the Clavien–Dindo classification. [file BJU-135-171-s001.docx]

### Supplementary material

Table S1

| **Visit** | **N = 97** |
| --- | --- |
| Number of pads at 6 weeks, n (%) |  |
| 0 | 62 (64) |
| 1 | 21 (22) |
| 2 | 8 (8.2) |
| 3 | 4 (4.1) |
| 4 | 1 (1.0) |
| 5 | 1 (1.0) |
| Number of pads at 3 months, n (%) |  |
| 0 | 78 (80) |
| 1 | 16 (16) |
| 2 | 1 (1.0) |
| 3 | 1 (1.0) |
| 4 | 1 (1.0) |
| Number of pads at 6 months, n (%) |  |
| 0 | 88 (91) |
| 1 | 7 (7.2) |
| 2 | 2 (2.1) |
| Number of pads 12 months, n (%) |  |
| 0 | 91 (94) |
| 1 | 4 (4.1) |
| 2 | 2 (2.1) |

Table S2

| **Variable** | **N = 97** |
| --- | --- |
| Post-operative complications, n (%) | 10 (10.3%) |
| Complication grade (Clavien-Dindo), n (%) |  |
| 1 | 5 (55.6%) |
| 2 | 4 (44.4%) |
| Description of complications: |  |
| Anastomosis leak | 2 |
| Postoperative ileus | 2 |
| Pelvic hematoma (managed conservatively) | 1 |
| Urinary retention | 1 |
| Urinary tract infection | 3 |
